# Confidence-Guided Adaptive Diffusion Network for Medical Image Classification

**DOI:** 10.3390/jimaging12020080

**Published:** 2026-02-14

**Authors:** Yang Yan, Zhuo Xie, Wenbo Huang

**Affiliations:** School of Computer Science and Technology, Changchun Normal University, Changchun 130032, China

**Keywords:** medical image classification, diffusion models, confidence-guided noise injection, multi-scale semantic modeling, prior-guided diffusion

## Abstract

Medical image classification is a fundamental task in medical image analysis and underpins a wide range of clinical applications, including dermatological screening, retinal disease assessment, and malignant tissue detection. In recent years, diffusion models have demonstrated promising potential for medical image classification owing to their strong representation learning capability. However, existing diffusion-based classification methods often rely on oversimplified prior modeling strategies, which fail to adequately capture the intrinsic multi-scale semantic information and contextual dependencies inherent in medical images. As a result, the discriminative power and stability of feature representations are constrained in complex scenarios. In addition, fixed noise injection strategies neglect variations in sample-level prediction confidence, leading to uniform perturbations being imposed on samples with different levels of semantic reliability during the diffusion process, which in turn limits the model’s discriminative performance and generalization ability. To address these challenges, this paper proposes a Confidence-Guided Adaptive Diffusion Network (CGAD-Net) for medical image classification. Specifically, a hybrid prior modeling framework is introduced, consisting of a Hierarchical Pyramid Context Modeling (HPCM) module and an Intra-Scale Dilated Convolution Refinement (IDCR) module. These two components jointly enable the diffusion-based feature modeling process to effectively capture fine-grained structural details and global contextual semantic information. Furthermore, a Confidence-Guided Adaptive Noise Injection (CG-ANI) strategy is designed to dynamically regulate noise intensity during the diffusion process according to sample-level prediction confidence. Without altering the underlying discriminative objective, CG-ANI stabilizes model training and enhances robust representation learning for semantically ambiguous samples.Experimental results on multiple public medical image classification benchmarks, including HAM10000, APTOS2019, and Chaoyang, demonstrate that CGAD-Net achieves competitive performance in terms of classification accuracy, robustness, and training stability. These results validate the effectiveness and application potential of confidence-guided diffusion modeling for two-dimensional medical image classification tasks, and provide valuable insights for further research on diffusion models in the field of medical image analysis.

## 1. Introduction

Medical image classification plays a crucial role in diagnostic imaging by automatically categorizing medical images according to predefined clinical criteria, thereby providing effective support for clinical decision-making [1]. At present, this technology has been widely applied across various medical domains, including dermoscopic image classification for skin disease diagnosis [2], histopathological image analysis for colorectal lesion recognition, and automated lung CT image detection for pneumonia and other thoracic disease screening [3].

Despite significant progress in recent years, the performance of medical image classification systems remains highly dependent on data quality and task-specific conditions. In real-world clinical settings, challenges such as data heterogeneity, class imbalance, imaging noise, and limited annotated samples often coexist, leading to unstable predictions or imbalanced confidence estimates and thereby hindering reliable clinical deployment [4]. Consequently, improving the robustness, stability, and reliability of medical image classification models under complex and uncertain conditions has become one of the most critical challenges in this field.

Early medical image classification approaches primarily relied on convolutional neural networks (CNNs) for feature extraction and discriminative modeling, particularly following the success of LeNet [5]. Classic architectures such as AlexNet [6] further accelerated the adoption of deep learning in medical imaging and inspired numerous applications, including MRI-based disease classification tasks [7]. Through hierarchical convolution and pooling operations, CNNs progressively learn representations ranging from low-level structural patterns to high-level semantic features. However, in medical image classification tasks characterized by limited labeled data, high inter-class similarity, or subtle pathological differences, conventional CNNs often struggle to adequately capture intrinsic uncertainty within samples, which may result in fluctuating predictions and limited stability.

To alleviate the challenges associated with data scarcity and high annotation costs in medical imaging, transfer learning has been widely adopted in medical image classification tasks [8,9]. Although transfer learning can improve classification performance in small-sample scenarios to some extent, the substantial differences in imaging mechanisms and semantic structures between natural and medical images often prevent pretrained features from accurately representing disease-related discriminative patterns and uncertainty characteristics. As a result, model generalization ability and prediction reliability remain constrained.

Building upon these developments, attention mechanisms [10] have been introduced into medical image classification to enhance discriminative performance by strengthening the model’s focus on key regions and informative features. For example, MEDUSA [11] employs multi-scale self-attention to model cross-scale contextual dependencies, leading to improved feature representation under complex structural conditions. More recently, state-space-model-based architectures such as Mamba have also been explored for medical image analysis, offering efficient long-range dependency modeling and improved scalability [12]. Nevertheless, most of these approaches enhance feature discrimination in a deterministic manner and lack explicit modeling of prediction confidence and uncertainty. When confronted with ambiguous or highly similar pathological patterns, they may still produce overconfident or unstable predictions.

Beyond the aforementioned approaches, various deep learning strategies have been proposed to address issues such as class imbalance, noisy annotations, and complex visual patterns in medical image classification. For instance, multi-task learning (MTL) [13] and margin-based discriminative learning methods such as LDAM [14] mitigate performance degradation caused by class imbalance through class-aware optimization objectives. Contrastive learning (CL) methods [15] enhance representation discriminability by enforcing stronger inter-feature constraints, while ProCo [16] improves robustness under noisy labels via prototype-based contrastive objectives. In addition, structured or sample-selection-driven approaches, including Co-teaching [17], INCV [18], and OUSM [19], improve classification performance by explicitly modeling class relationships or filtering out anomalous samples. However, most of these methods rely on fixed feature extraction pipelines or static optimization strategies and lack explicit modeling of the dynamic evolution of representations during training, particularly from a generative or reconstruction perspective that can systematically characterize sample uncertainty.

From another perspective, generative models have also been introduced to mitigate data scarcity in medical image classification. Generative adversarial networks (GANs) [20] perform data augmentation by synthesizing training samples, thereby improving model generalization to some extent [21,22,23]. Nevertheless, GAN-based approaches often suffer from training instability, mode collapse, and limited sample diversity. Moreover, GANs are primarily used as auxiliary data generation tools and are difficult to integrate directly into the classification decision process or prediction confidence modeling.

In recent years, diffusion models [24] have emerged as a powerful generative modeling framework and have gradually been introduced into medical image analysis due to their stability and expressive capacity in modeling complex data distributions [25,26]. Through forward noise injection and iterative reverse denoising processes, diffusion models can learn probabilistic latent representations. Recent studies have demonstrated that diffusion mechanisms also exhibit potential advantages in discriminative tasks such as medical image classification. For example, Chen et al. [27] systematically analyzed the discriminative capability of diffusion models from a representation learning perspective, while DiffMIC, proposed by Yang et al. [28], incorporates diffusion mechanisms into medical image classification to suppress random noise and artifacts through progressive denoising, enabling more robust semantic feature extraction. More recent studies further suggest that conditional diffusion models can simultaneously provide classification predictions, uncertainty estimates, and interpretability.

In addition, several uncertainty-aware and noise-robust learning approaches have been proposed for medical image classification. For instance, HSA-NRL [29] introduces a hard-sample-aware learning mechanism that effectively alleviates the adverse effects of label noise during training. Other recent works have explored uncertainty-aware learning strategies to improve reliability under ambiguous or low-quality inputs [30]. Although these methods have achieved certain improvements in robustness, they generally focus on enhancing discriminative performance under fixed feature extraction or static noise modeling strategies, lacking joint modeling of hierarchical semantic stability and sample-adaptive noise regulation throughout the diffusion process.

Although diffusion models have demonstrated significant potential for medical image classification, existing diffusion-based approaches still face two fundamental challenges. First, during the progressive denoising process, the semantic hierarchy of feature representations evolves across diffusion stages; however, most existing methods lack explicit mechanisms to enforce cross-stage semantic consistency, which may result in semantic drift and feature instability, as critically discussed in prior work [31]. Second, many approaches directly adopt fixed noise scheduling strategies originally developed for generative tasks. Such strategies prioritize distribution coverage rather than discriminative optimization and therefore overlook the pronounced heterogeneity in sample difficulty and prediction confidence inherent in medical images, potentially leading to excessive semantic corruption in early training stages and optimization instability.

Beyond these challenges, current diffusion-based classifiers commonly rely on structurally simplistic prior representations and static noise regulation mechanisms, limiting their capacity to capture hierarchical semantic relationships and adapt to heterogeneous sample uncertainty. Collectively, these limitations point to a critical gap in current research: the absence of a unified framework that simultaneously enforces hierarchical semantic consistency while enabling confidence-aware noise adaptation.

To bridge this gap, we propose a confidence-guided adaptive diffusion-based medical image classification framework, termed CGAD-Net. At the architectural level, a Hierarchical Pyramid Context Modeling (HPCM) module and an Intra-Scale Dilated Convolution Refinement (IDCR) module are introduced to enhance semantic stability during the denoising process by achieving cross-level semantic alignment and intra-scale structural refinement, respectively. At the optimization level, a Confidence-Guided Adaptive Noise Injection (CG-ANI) strategy dynamically regulates noise intensity according to sample-level prediction confidence, thereby alleviating the mismatch between fixed noise schedules and discriminative learning objectives. Notably, CG-ANI is designed as a training stabilization mechanism rather than a direct performance enhancement module, enabling improved convergence stability without introducing additional inference overhead.

The main contributions of this work are summarized as follows:

1. We identify a critical limitation in existing diffusion-based medical image classification methods, namely the lack of explicit modeling of hierarchical semantic consistency during the denoising process, and propose two structural modules—Hierarchical Pyramid Context Modeling (HPCM) and Intra-Scale Dilated Convolution Refinement (IDCR)—to stabilize diffusion feature representations.
2. We propose a Confidence-Guided Adaptive Noise Injection (CG-ANI) strategy that effectively alleviates the mismatch between fixed noise scheduling and discriminative learning objectives, thereby improving training stability in diffusion-based classification.
3. By integrating structural prior modeling with an optimization stabilization mechanism, we construct a unified diffusion-based medical image classification framework, termed CGAD-Net, which enables stable feature learning across different denoising stages.
4. Extensive experiments conducted on multiple medical image classification benchmarks suggest that CGAD-Net achieves competitive performance relative to existing state-of-the-art methods in terms of classification accuracy, robustness, and cross-dataset generalization.

## 2. Methods

**Figure 1:**
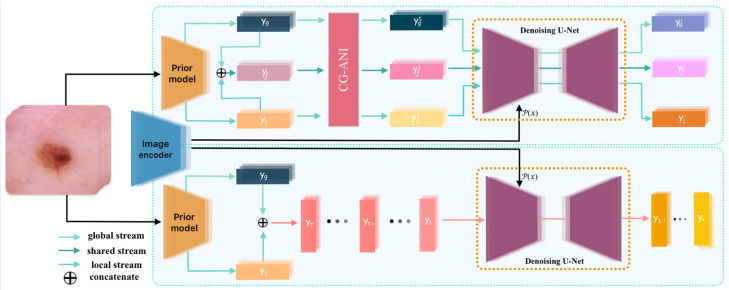
Architecture of the proposed CGAD-Net framework.

The overall architecture of the proposed Confidence-Guided Adaptive Diffusion Network (CGAD-Net) is illustrated in Figure 1. CGAD-Net is formulated as a unified diffusion-based optimization framework rather than a sequential pipeline of independent modules. As shown in Figure 1, the framework consists of three tightly coupled components: hierarchical prior modeling, confidence-guided adaptive noise injection, and diffusion-based reconstruction. These components are jointly optimized under a shared training objective, enabling coherent semantic representation learning in an end-to-end manner.

Given an input image *x*, two parallel pathways are first established. The image is processed by an image encoder to obtain a latent representation ρ(x), which serves as the conditional signal for diffusion-based reconstruction. Simultaneously, the image is fed into a prior model to extract multi-level semantic priors.

Specifically, the prior model generates a *global prior feature* $y_{g}$, which captures coarse contextual semantics across the entire image, and a *local prior feature* $y_{l}$, which focuses on fine-grained structural patterns within potential regions of interest. These two priors are further fused through feature concatenation to form a *fused prior representation* $y_{f}$, enabling the joint encoding of global contextual information and localized morphological details.

To enhance robustness under heterogeneous data conditions, a Confidence-Guided Adaptive Noise Injection (CG-ANI) mechanism is applied to the prior representations. At diffusion step *t*, prediction confidence is estimated from the classifier and used to modulate the noise magnitude. Confidence-aware noise is independently injected into the global prior $y_{g}$, local prior $y_{l}$, and fused prior $y_{f}$, producing the noisy representations $y_{g}^{t}$, $y_{l}^{t}$, and $y_{f}^{t}$, respectively. This adaptive perturbation process preserves discriminative semantics for high-confidence samples while promoting more robust feature exploration for uncertain cases.

The noisy priors are then jointly conditioned on the latent encoding ρ(x) and passed into a U-Net-based denoising network. The network is trained to predict the injected noise and reconstruct semantically meaningful representations, yielding the denoised features ${\hat{y}}_{g}$, ${\hat{y}}_{l}$, and ${\hat{y}}_{f}$ for each branch. To further stabilize reconstruction, each denoised feature is regularized against its corresponding clean prior through a reconstruction constraint.

All components of CGAD-Net are collaboratively optimized within a unified objective, ensuring that prior modeling and diffusion-based reconstruction evolve synergistically rather than sequentially. During inference, the trained denoising network performs the reverse diffusion process, progressively removing noise to recover the clean target representation $y_{0}$. This noise-free representation serves as the final discriminative feature for downstream medical image classification.

To improve methodological transparency and emphasize the unified nature of the framework, the complete end-to-end training procedure is summarized in Algorithm 1. This formulation clarifies the interactions among hierarchical priors, confidence-aware noise modulation, and diffusion reconstruction, highlighting how they are integrated into a cohesive optimization process.
**Algorithm 1** End-to-End Unified Training Procedure of CGAD-Net**Require:** Training dataset $D={\left\{\left(x_{i},y_{i}\right)\right\}}_{i=1}^{N}$  1:**for** each training iteration **do**  2:    Extract hierarchical prior features via HPCM and IDCR.  3:    Construct fused semantic prior representations.  4:    Compute prediction confidence from the classifier.  5:    Inject confidence-guided adaptive noise into prior features.  6:    Perform diffusion-based denoising with a U-Net backbone.  7:    Jointly optimize the unified training objective.  8:**end for**        **return** the trained CGAD-Net model.


### 2.1. Prior Module Design

**Figure 2:**
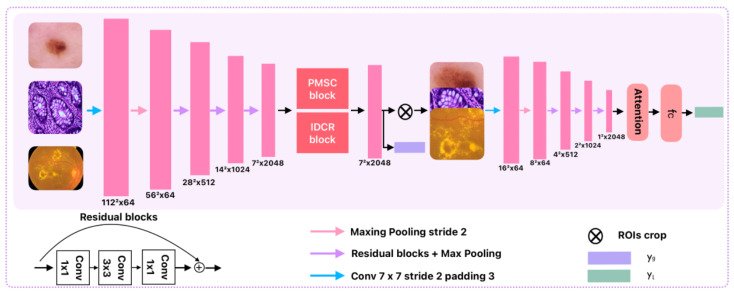
Overview of the architecture of the proposed Prior Model within CGAD-Net.

In conditional diffusion-based medical image classification frameworks, prior representations serve as stable semantic constraints for both the forward diffusion process and the reverse denoising reconstruction process, thereby supporting discriminative feature reconstruction. To simultaneously capture the multi-scale contextual information and fine-grained local structural characteristics that are ubiquitous in medical images, this work designs a structure-aware prior modeling module that jointly models global semantic information and local structural cues. As illustrated in Figure 2, the input image is first processed by a standard convolutional layer to extract low-level feature representations, which are then fed into a downsampling encoder path composed of alternating residual blocks and max-pooling layers. By progressively expanding the receptive field, this encoder performs hierarchical semantic abstraction and generates high-dimensional feature representations with strong global perceptual capability, providing a foundational representation for subsequent prior feature modeling.

Building upon the above design, to explicitly address two critical semantic instability issues encountered in diffusion-based classification—namely cross-scale semantic misalignment and intra-scale structural inconsistency—this work introduces two complementary feature modeling modules: the Hierarchical Pyramid Context Modeling (HPCM) module and the Intra-Scale Dilated Convolution Refinement (IDCR) module.

**Figure 3:**
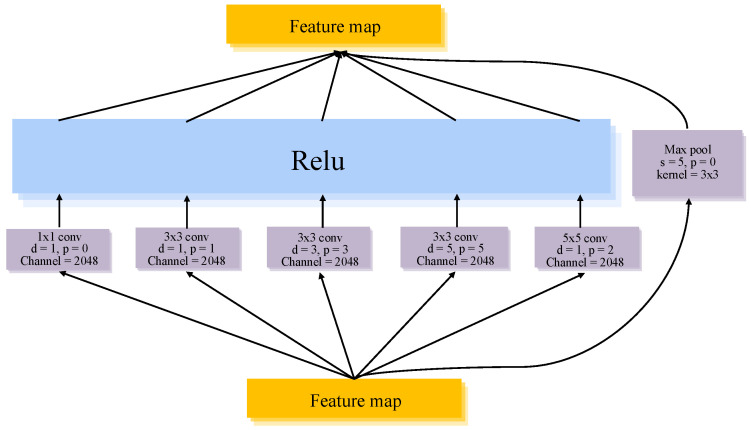
HPCM Module.

The HPCM module is designed to enhance cross-scale semantic alignment. As illustrated in Figure 3, this module constructs a hierarchical context modeling pathway by integrating standard convolutions with dilated convolutions of varying dilation rates, thereby enabling the simultaneous capture of local details and long-range dependencies within a unified feature space. By explicitly aggregating contextual information across multiple receptive field scales, HPCM stabilizes the semantic abstraction process among features at different scales and strengthens the model’s capability to perceive global pathological patterns.

**Figure 4:**
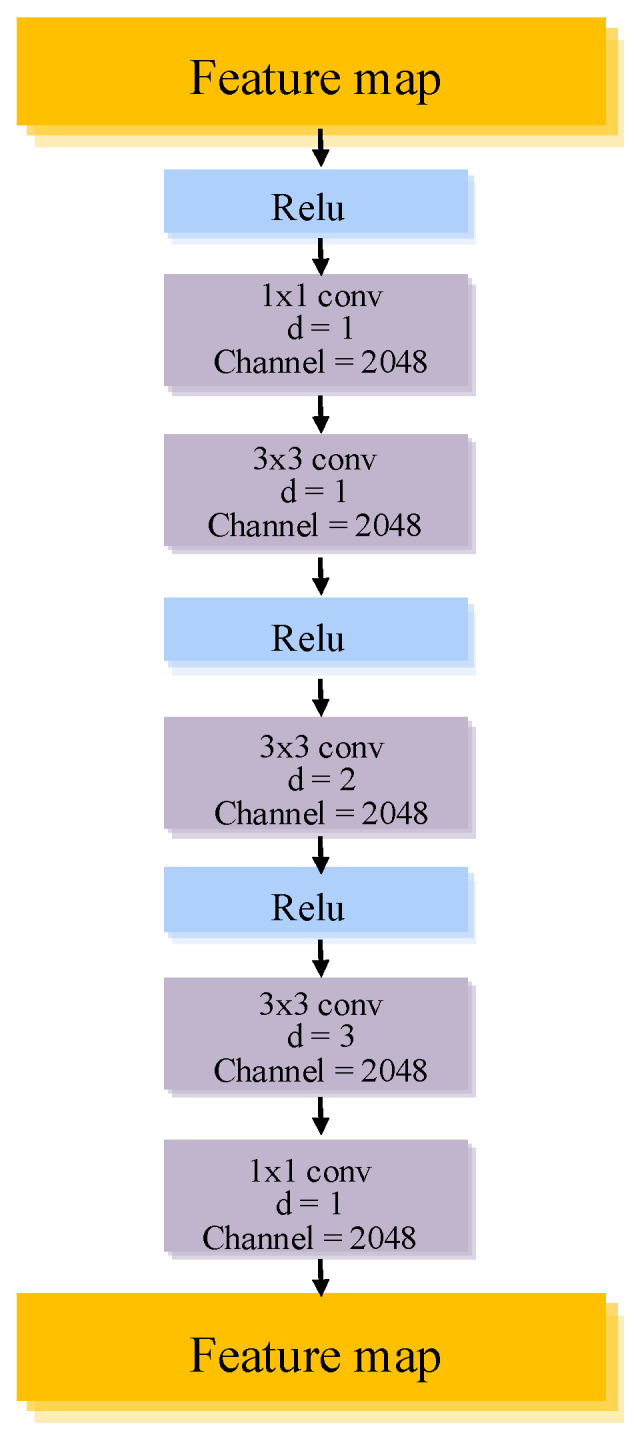
IDCR Module.

Complementary to HPCM, the IDCR module focuses on refining and stabilizing feature representations within the same scale. As shown in Figure 4, the IDCR module is presented as a more explicit architectural formulation of the intra-scale feature refinement strategy employed in CGAD-Net. This structure more accurately reflects the implementation used throughout the experiments, where progressively dilated convolutions are stacked to expand the receptive field while preserving spatial resolution. IDCR sequentially stacks dilated convolutions with progressively increasing dilation rates, allowing the receptive field to be gradually expanded while preserving the original feature resolution. Through progressive feature refinement, this module effectively suppresses redundant or noisy activations and enhances structural consistency within the same semantic level, which is particularly critical for distinguishing subtle and highly similar pathological patterns in medical images.

The output features of HPCM and IDCR are concatenated along the channel dimension to form a global prior representation with enriched semantic diversity and improved structural consistency. Guided by this global prior, the model generates discriminative spatial responses to localize potential regions of interest (ROI). The cropped ROIs are subsequently fed into a local feature extraction pathway composed of deep residual blocks and pooling layers to further aggregate fine-grained structural information. In addition, a channel attention mechanism is incorporated to emphasize key pathological regions, and the resulting features are finally mapped through a fully connected layer to obtain the prior information representation.

The architectural design of the proposed prior modeling module is guided by the objective of achieving semantically stable feature representations throughout the diffusion process rather than simply increasing model complexity. Unlike heuristic module stacking, each component is introduced to address a specific limitation observed in diffusion-based medical image classification.

In particular, hierarchical semantic inconsistency across receptive fields can lead to unstable feature evolution during progressive denoising, while insufficient structural refinement within the same scale may reduce discriminative sensitivity to subtle pathological variations. To mitigate these issues in a principled manner, we explicitly decompose prior modeling into two complementary objectives: cross-scale semantic alignment and intra-scale structural stabilization. This design philosophy ensures that the proposed framework operates as a cohesive representation learning mechanism rather than a sequential pipeline of independent modules.

### 2.2. Confidence-Guided Adaptive Noise Injection Strategy

To provide further theoretical intuition for the proposed strategy, it is important to note that the objective of noise injection in diffusion models is not merely to corrupt the input, but to enable the model to learn stable representations by reconstructing signals from stochastic perturbations. From a probabilistic perspective, controlled noise injection encourages the model to explore a smoother data manifold and reduces sensitivity to spurious correlations, thereby improving generalization.

In conventional diffusion formulations, the noise magnitude is determined solely by the diffusion timestep, implicitly assuming homogeneous uncertainty across samples. However, medical images often exhibit substantial variability in semantic reliability. For example, images with blurred lesion boundaries or low contrast typically induce higher predictive uncertainty than images with clearly distinguishable pathological patterns. Treating these heterogeneous samples with an identical noise schedule may either over-corrupt reliable representations or insufficiently regularize uncertain ones.

The proposed Confidence-Guided Adaptive Noise Injection (CG-ANI) strategy addresses this limitation by introducing prediction confidence as a proxy for semantic reliability. Specifically, lower confidence is interpreted as an indicator of higher uncertainty, motivating stronger perturbation to prevent premature convergence toward unreliable feature configurations. Conversely, higher confidence suggests that the current representation already captures stable discriminative cues, and therefore excessive noise injection may unnecessarily disrupt the learned semantics.

To formalize this intuition, we present a simplified confidence-guided perturbation formulation during training. Given an input sample with ground-truth label $y_{0}$, the model’s current prediction *y*, and the corresponding confidence score *c*, the perturbed representation is defined as:

(1) $$y_{t}=t\cdot y+\left(1-t\right)\cdot y_{0}+\left(1-c\right)\cdot \epsilon ,$$

where t∈[0,1] denotes a normalized diffusion timestep, *c* represents the model’s prediction confidence, and ϵ∼N(0,I) is Gaussian noise.

This formulation reflects an uncertainty-aware regularization mechanism. When the prediction confidence is low (c→0), the increased contribution of stochastic noise encourages broader exploration of the representation space, reducing the risk of overfitting to unreliable predictions. In contrast, when the confidence is high, attenuating the noise term helps preserve critical semantic structures while maintaining stable optimization trajectories.

From an optimization standpoint, this adaptive perturbation can be interpreted as a form of confidence-weighted regularization that dynamically balances representation stability and stochastic exploration. Rather than adding noise solely to be removed, the process promotes the learning of noise-invariant features that remain consistent under controlled perturbations, thereby improving robustness in the presence of semantic ambiguity.

In practical implementation, the above intuitive formulation is extended into an adaptive noise injection strategy with a rigorous probabilistic interpretation and embedded within the standard forward diffusion modeling framework. At diffusion step *t*, the forward diffusion process is defined as:

(2) $$y_{t}=\sqrt{{\bar{\alpha }}_{t}}\cdot y_{0}+\sqrt{1-{\bar{\alpha }}_{t}}\cdot \left(1-c\right)\cdot \epsilon +\left(1-\sqrt{{\bar{\alpha }}_{t}}\right)\cdot \left({\hat{y}}_{g}+{\hat{y}}_{l}\right),$$

Here, ${\bar{\alpha }}_{t}$ denotes the cumulative noise decay factor at diffusion step *t*, $y_{0}$ represents the clean target representation, and ϵ∼N(0,I) is Gaussian noise. The term $\left({\hat{y}}_{g}+{\hat{y}}_{l}\right)$ denotes the guidance representation constructed from the global and local prior branches, which imposes semantic constraints during the noise injection process. The confidence score *c* acts as a modulation factor that adaptively scales the noise intensity, such that uncertain samples are subjected to stronger perturbations, while the noise applied to high-confidence samples is effectively attenuated.

During the reverse denoising stage, a confidence-aware denoising mechanism is further introduced to enable adaptive sampling based on prediction reliability. Samples with lower confidence correspond to higher semantic uncertainty and therefore retain more noise during denoising to promote robust representation learning, whereas samples with high confidence undergo stronger noise suppression to recover clean target representations. Accordingly, the denoising operation at diffusion step *t* is defined as:

(3) $${\hat{y}}_{0}=\frac{1}{\sqrt{{\bar{\alpha }}_{t}}}\left(y_{t}-\left(1-\sqrt{{\bar{\alpha }}_{t}}\right)\cdot {\bar{y}}_{T}-\left(1-c\right)\cdot \sqrt{1-{\bar{\alpha }}_{t}}\cdot \epsilon_{\theta }\right),$$

Here, ${\hat{y}}_{0}$ denotes the estimated clean representation, ${\bar{y}}_{T}$ represents the sample mean after *T* diffusion steps and serves as the prior center of the data distribution, and $\epsilon_{\theta }$ is the noise term predicted by the denoising network. The factor (1−c) is used to adaptively modulate the denoising strength, thereby enabling differentiated denoising strategies across samples with varying confidence levels.

After obtaining ${\hat{y}}_{0}$, a reparameterization-based sampling scheme is employed to generate the sample at the previous diffusion time step. The corresponding reverse transition process is defined as:

(4) $$y_{t-1}=\mu_{t}\left({\hat{y}}_{0},y_{t},y_{g}\right)+\sqrt{{\tilde{\beta }}_{t}}\cdot \epsilon ,$$

Here, $\mu_{t}\left(\cdot \right)$ denotes the Bayesian posterior mean conditioned on the estimated clean representation ${\hat{y}}_{0}$, the current noisy representation $y_{t}$, and the global prior $y_{g}$; ${\tilde{\beta }}_{t}$ represents the posterior variance at diffusion step *t*; and ϵ∼N(0,I) is the reparameterization noise.

By incorporating confidence guidance into the reverse diffusion process, the stochasticity of sampling is adaptively regulated: samples with high confidence correspond to smaller posterior variance, leading to faster convergence during sampling, whereas samples with low confidence retain higher randomness to encourage sufficient exploration of the latent semantic space. This design enables a dynamic balance between stability and robustness.

Within the CG-ANI mechanism, the confidence score *c* is used to jointly regulate both noise injection strength and denoising intensity. In practice, *c* is computed as the maximum posterior probability output by the classification head:

(5) $$c=\max_{k}p_{\theta }\left(y=k|x\right),$$

Here, $p_{\theta }\left(\cdot \right)$ denotes the softmax output of the classifier parameterized by θ. This definition provides a computationally efficient measure of prediction reliability without introducing additional model components or inference overhead. To ensure training stability, the confidence score is detached from the computation graph when used for noise modulation.

It should be emphasized that the confidence score *c* is not a strict uncertainty estimation, but rather a lightweight proxy for semantic reliability that reflects the model’s relative certainty in its current prediction. During training, the ground-truth label $y_{0}$ is available and can be used to construct the forward diffusion process defined in Equation (2); during inference, where ground-truth labels are unavailable, the model prediction is used in place of $y_{0}$, and the confidence score is computed entirely based on the predicted output. This design ensures that CG-ANI does not rely on any prior or oracle information at test time.

Overall, the training and inference procedures follow the standard diffusion modeling framework, with the CG-ANI strategy consistently applied during both forward noise injection and reverse denoising. By dynamically modulating noise intensity and sampling stochasticity based on sample-level confidence, the proposed method adaptively balances model stability and robustness across heterogeneous medical image samples.

### 2.3. Loss Function

To train the proposed diffusion-based classification framework in a stable and robust manner, we define a unified training objective that consists of a confidence-aware denoising regression loss, a multi-view regularization term, and a lightweight auxiliary supervision mechanism. These components jointly facilitate effective noise prediction, semantic alignment across priors, and reliable class discrimination.

#### 2.3.1. Adaptive Denoising Regression Loss

The primary training objective of CGAD-Net is to learn an accurate noise prediction function for the reverse diffusion process. During training, an adaptively noise-corrupted target representation $y_{t}$ is constructed based on the confidence-guided forward diffusion formulation:

(6) $$y_{t}=\sqrt{{\bar{\alpha }}_{t}}\cdot y_{0}+\sqrt{1-{\bar{\alpha }}_{t}}\cdot \left(1-c\right)\cdot \epsilon +\left(1-\sqrt{{\bar{\alpha }}_{t}}\right)\cdot \left({\hat{y}}_{g}+{\hat{y}}_{l}\right),$$

where $y_{0}$ denotes the target clean representation, *c* is the confidence score of the current sample, and ϵ∼N(0,I) is Gaussian noise. The term $\left({\hat{y}}_{g}+{\hat{y}}_{l}\right)$ represents the guided prior constructed from the global and local prior branches.

Conditioned on the input image *x*, the noisy target $y_{t}$, the diffusion step *t*, and the guided prior, the denoising network is trained to predict the injected noise. The denoising regression loss is defined as

(7) $$L_{\mathrm{denoise}}={∥\epsilon -\hat{\epsilon }\left(x,y_{t},t,{\hat{y}}_{g}+{\hat{y}}_{l}\right)∥}^{2},$$

which serves as the core optimization objective for learning the reverse diffusion dynamics.

#### 2.3.2. Multi-View Maximum Mean Discrepancy Regularization

To encourage consistent semantic representations across different prior views, we introduce a multi-view Maximum Mean Discrepancy (MMD) regularization term. Specifically, two conditional denoising paths are constructed based on the global prior $y_{g}$ and the local prior $y_{l}$, producing the corresponding noise predictions ${\hat{\epsilon }}_{g}$ and ${\hat{\epsilon }}_{l}$. The distributional discrepancy between these predictions and the ground-truth noise ϵ is measured as

(8) $$L_{\mathrm{MMD}}=\mathrm{MMD}\left({\hat{\epsilon }}_{g},\epsilon \right)+\mathrm{MMD}\left({\hat{\epsilon }}_{l},\epsilon \right),$$

where the MMD distance is defined by

(9) $$\mathrm{MMD}\left(x,y\right)=E\left[k\left(x,x^{\prime }\right)\right]+E\left[k\left(y,y^{\prime }\right)\right]-2E\left[k\left(x,y\right)\right],$$

and k(·,·) denotes a Gaussian kernel. This regularization term serves to align the noise prediction distributions from different prior-guided views, thereby promoting semantic consistency during training.

#### 2.3.3. Periodic Auxiliary Supervision

To further stabilize training and provide additional class-discriminative guidance, we introduce a periodically activated auxiliary classifier branch. This auxiliary pathway predicts the image label $y_{0}$ based on the input image and is trained using the standard cross-entropy loss:

(10) $$L_{\mathrm{aux}}=\mathrm{CE}\left(f_{\mathrm{aux}}\left(x\right),y_{0}\right),$$

where $f_{\mathrm{aux}}\left(\cdot \right)$ denotes the auxiliary classifier and CE(·) represents the cross-entropy loss. The auxiliary branch is activated at fixed training intervals and acts as a lightweight supervision signal to complement the diffusion-based objective, particularly during early training stages.

#### 2.3.4. Overall Training Objective

The final training objective is defined as a weighted combination of the above loss components:

(11) $$L_{\mathrm{total}}=L_{\mathrm{denoise}}+\lambda \cdot \left(L_{\mathrm{MMD}}+L_{\mathrm{aux}}\right),$$

where λ is a balancing coefficient that controls the contribution of the regularization and auxiliary terms. In all experiments, λ is empirically set to 0.5.

## 3. Results

### 3.1. Datasets and Evaluation

To comprehensively evaluate the generalization capability of CGAD-Net under heterogeneous medical imaging conditions, we select three publicly available benchmark datasets: HAM10000, APTOS2019, and Chaoyang. These datasets were deliberately chosen because they exhibit substantial diversity in imaging modality, class distribution, and clinical characteristics, thereby providing a challenging and representative testbed for diffusion-based classification models.

The HAM10000 dataset consists of 10,015 dermoscopic images categorized into seven common skin lesion classes, including Actinic Keratoses and Intraepithelial Carcinoma (AKIEC), Basal Cell Carcinoma (BCC), Benign Keratosis-like Lesions (BKL), Dermatofibroma (DF), Melanocytic Nevi (NV), Melanoma (MEL), and Vascular Lesions (VASC). The APTOS2019 dataset comprises 3662 color fundus images annotated with five severity levels of diabetic retinopathy. The Chaoyang dataset contains 6160 histopathological images covering four types of colorectal tissue.

For all datasets, CGAD-Net is trained and evaluated strictly following the original training and testing splits provided by the dataset authors to ensure fair and reproducible performance comparison. Quantitative evaluation is conducted using two widely adopted metrics, namely classification accuracy (Accuracy) and F1-score.

The comparative results between CGAD-Net and representative state-of-the-art (SOTA) methods are reported in Table 1 and Table 2. The performance of the comparison methods is taken directly from their original publications under the respective experimental settings. Although the experimental protocols across different methods may not be fully identical, these comparisons are intended to provide a reference for overall performance trends.

### 3.2. Implementation Details

During both training and testing, a systematic data preprocessing and augmentation pipeline is designed to address data scarcity and high sample diversity in medical imaging, thereby enhancing model generalization and mitigating overfitting. In the training stage, all input images are first uniformly resized to a fixed resolution and center-cropped to ensure spatial consistency while preserving regions of interest. Subsequently, a series of spatial data augmentation operations is applied, including random rotations within ±30°, horizontal flipping, and vertical flipping, to simulate common geometric variations encountered in clinical imaging. Finally, all images are normalized using channel-wise means of [0.485, 0.456, 0.406] and standard deviations of [0.229, 0.224, 0.225], consistent with the statistics used by ImageNet-pretrained models.

During the testing stage, all stochastic data augmentation operations are removed, and only deterministic resizing and center cropping are retained to ensure evaluation stability and reproducibility. All experiments are implemented using the PyTorch framework and conducted on a single NVIDIA A100 GPU. The model is trained with a batch size of 64 for a total of 1000 epochs. The Adam optimizer is employed with an initial learning rate of $1\times 10^{-3}$. A cosine annealing learning rate scheduling strategy is applied during training, allowing the learning rate to gradually decay over the course of optimization and facilitating smooth convergence.

### 3.3. Performance Comparison with State-of-the-Art Methods

We conduct a systematic comparison between the proposed CGAD-Net and a set of representative state-of-the-art (SOTA) methods on three publicly available medical image classification benchmarks, namely HAM10000, APTOS2019, and Chaoyang. The comparison methods include both traditional convolutional neural network (CNN)-based classifiers and more recent approaches enhanced with diffusion or attention mechanisms. The reported results of the comparison methods are taken directly from their original publications. CGAD-Net is trained and evaluated following the official training and testing splits provided by each dataset, and all methods are compared under the same evaluation metrics to promote result comparability.

As shown in Table 1, CGAD-Net achieves an accuracy of 0.914 and an F1-score of 0.839 on the HAM10000 dataset, demonstrating competitive performance relative to the reported methods. Compared with the diffusion-based DiffMIC, CGAD-Net yields an absolute improvement of 0.9% in accuracy and 2.4% in F1-score. The relatively larger gain in F1-score may indicate improved sensitivity to minority classes or visually similar lesion categories, which are common in HAM10000. From a representation learning perspective, this behavior may be associated with the hierarchical prior modeling strategy, which promotes cross-scale semantic consistency and helps preserve discriminative cues during the diffusion denoising process. Nevertheless, further class-level analysis would be beneficial to more definitively characterize this effect.

On the APTOS2019 dataset, which is characterized by pronounced class imbalance and subtle inter-class differences, CGAD-Net achieves an accuracy of 0.855 and an F1-score of 0.675. Although the improvement in accuracy is relatively modest, the observed gain in F1-score suggests that the proposed framework may better accommodate underrepresented diabetic retinopathy grades. One possible explanation is that the confidence-guided noise injection mechanism reduces excessive semantic perturbation for high-confidence samples while maintaining sufficient stochastic exploration for uncertain cases, thereby supporting more stable feature learning under heterogeneous data conditions.

For the Chaoyang dataset, which consists of histopathological images with complex textures and substantial intra-class variability, CGAD-Net attains an accuracy of 0.878 and an F1-score of 0.830, as reported in Table 2. Relative improvements across both metrics suggest that the proposed framework is capable of capturing fine-grained morphological patterns while maintaining semantic coherence across diffusion stages. This capability may stem from the complementary interaction between hierarchical contextual modeling and intra-scale structural refinement, which together facilitate more robust feature representations for structurally complex medical images.

It is worth noting that, although the comparison methods were evaluated under their respective experimental protocols, CGAD-Net exhibits broadly consistent performance trends across all three heterogeneous benchmarks. Rather than indicating optimization toward a specific dataset or metric, these observations suggest that the proposed framework may contribute to improved representation stability during diffusion-based learning.

At the same time, the magnitude of improvement varies across datasets, implying that the benefits of CGAD-Net are likely influenced by data characteristics such as class distribution, structural complexity, and semantic ambiguity. This observation highlights the importance of evaluating diffusion-based classifiers under diverse clinical scenarios and suggests that future work could further investigate dataset-dependent behavior through finer-grained analyses.

**Figure 5:**
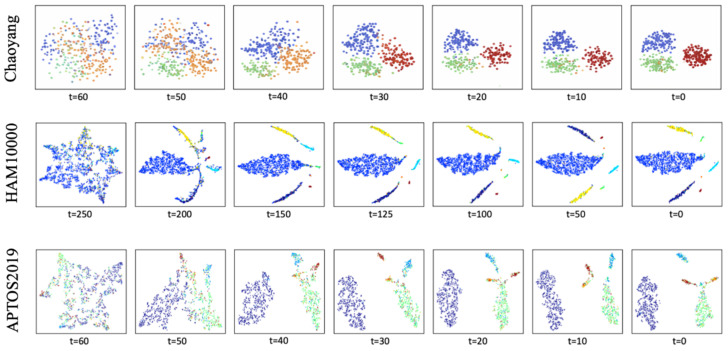
t-SNE visualization of denoised feature embeddings at different reverse diffusion time steps on the Chaoyang, HAM10000, and APTOS2019 datasets. Each point represents a sample, and different colors indicate different classes. As the reverse diffusion process progresses (t→0), the injected noise is gradually removed and the class distributions become increasingly separable.

To further analyze the effect of the proposed confidence-guided adaptive noise scheduling strategy during the reverse diffusion process, we perform t-SNE visualization of the feature representation evolution across different diffusion time steps, as illustrated in Figure 5. The experiments are conducted on the Chaoyang, HAM10000, and APTOS2019 datasets. For each dataset, intermediate denoised feature embeddings from multiple reverse diffusion stages are extracted and projected into a two-dimensional space, where different colors correspond to different class labels.

It can be observed that at larger diffusion time steps (e.g., *t* = 60 or *t* = 250), feature embeddings from different classes are highly intermixed, indicating that the representations at this stage are predominantly dominated by strong noise perturbations. As the reverse diffusion process progresses, noise is gradually removed and the feature distributions begin to evolve in an ordered manner along semantically meaningful directions. When the diffusion time step approaches zero, samples belonging to the same class gradually form compact clusters, while the discriminative boundaries between different classes become increasingly well defined.

These observations suggest that the reverse diffusion process is not merely a denoising operation, but also a progressive discriminative feature refinement procedure. When combined with the proposed confidence-guided noise scheduling strategy, the model is able to retain an appropriate level of stochasticity for semantically uncertain samples during intermediate diffusion stages, while allowing high-confidence samples to converge more rapidly toward stable and discriminative regions in the feature space. This confidence-based adaptive evolution mechanism contributes to improved class separability and overall robustness of the final classification results.

### 3.4. Ablation Study

To systematically analyze the contribution of each proposed component to the overall performance of CGAD-Net, comprehensive ablation experiments are conducted on the HAM10000 dataset. A controlled, stepwise ablation strategy is adopted, in which different modules are progressively enabled to evaluate both their individual effects and their joint impact on model behavior.

Notably, the baseline model (B0) follows the conventional fixed noise scheduling strategy commonly used in diffusion-based classification frameworks. Building upon this baseline, the proposed Confidence-Guided Adaptive Noise Injection (CG-ANI), the Hierarchical Pyramid Context Modeling (HPCM) module, and the Intra-Scale Dilated Convolution Refinement (IDCR) module are incrementally incorporated. This design enables explicit isolation of the optimization-level effect introduced by CG-ANI, as well as assessment of the complementary contributions of the structural modules.

The HAM10000 dataset is selected due to its relatively large number of classes, sufficient sample size, and high task complexity, providing a representative and challenging benchmark for component-level effectiveness analysis.

As reported in Table 3, the baseline model (B0), which adopts a fixed noise scheduling strategy, achieves an accuracy of 0.905, an F1-score of 0.815, and a Kappa coefficient of 0.805 on the HAM10000 dataset.

When the fixed schedule is replaced with the proposed CG-ANI while keeping the backbone architecture unchanged (B1), the model exhibits measurable improvements in both accuracy (+0.5%) and F1-score (+2.9%), with the Kappa coefficient increasing to 0.815. Because this comparison isolates the effect of noise scheduling from architectural modifications, the observed gains can be primarily attributed to confidence-guided noise modulation. These results suggest that adaptive noise regulation improves optimization stability and promotes more reliable feature learning, particularly for semantically ambiguous or low-confidence samples, rather than directly strengthening the discriminative objective.

With the further incorporation of the HPCM module (B2), the model accuracy increases to 0.912 and achieves the highest F1-score of 0.845. Compared with B1, the marginal gain in accuracy is relatively limited, whereas the more noticeable improvement in F1-score suggests enhanced discrimination capability for minority classes and visually similar categories. This observation is consistent with the design objective of HPCM, which aims to strengthen contextual modeling consistency during the diffusion denoising process through hierarchical semantic alignment across multiple receptive fields. Finally, when CG-ANI, HPCM, and the Intra-Scale Dilated Convolution Refinement (IDCR) module are jointly incorporated into the full model, CGAD-Net attains the highest overall accuracy of 0.914 and the highest Kappa coefficient of 0.825. Although its F1-score is slightly lower than that of B2, this phenomenon can be attributed to the differing optimization emphasis introduced by IDCR. Specifically, while HPCM primarily enhances fine-grained class separability, IDCR focuses on stabilizing intra-scale feature representations and reducing prediction variance across classes. This stabilization effect may not always translate into the highest F1-score but contributes to improved global decision consistency, as reflected by the increase in the Kappa coefficient.

It is worth noting that F1-score evaluates the balance between precision and recall on a per-class basis, whereas the Kappa coefficient reflects overall agreement between predictions and ground truth across the entire dataset. The observed trade-off therefore suggests that the IDCR module promotes more balanced decision boundaries rather than aggressively optimizing class-wise separability. Such behavior aligns with the design goal of improving representation stability within diffusion-based classification.

Although the ablation results demonstrate that each proposed module contributes to improved classification performance and decision consistency, it remains necessary to further clarify whether these performance gains genuinely arise from stabilization of the diffusion-based learning process, rather than from an implicit reduction in the difficulty of the optimization objective. In particular, the confidence-guided adaptive noise injection (CG-ANI) strategy explicitly modulates noise intensity based on prediction confidence, which naturally raises a critical question: does this strategy merely simplify the denoising task itself, thereby yielding superficial performance improvements?

**Figure 6:**
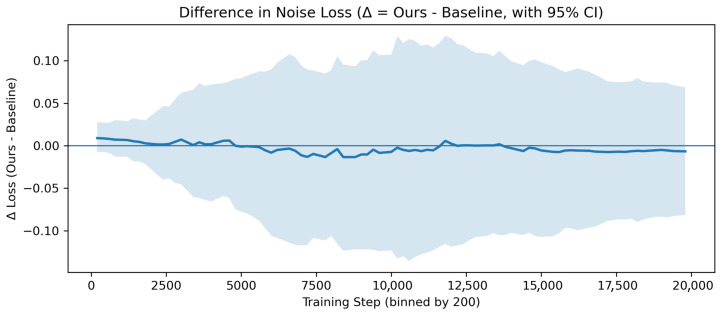
Difference in noise estimation loss between the CG-ANI-enhanced model and the baseline during training on the HAM10000 dataset. The solid curve represents the mean loss difference (Δ=Ours−Baseline). The shaded band indicates the 95% confidence interval (CI), reflecting the variability of the estimated difference obtained via bootstrap resampling across training steps.

To address this question, we further analyze the evolution of the noise estimation loss before and after introducing CG-ANI. As illustrated in Figure 6, throughout the entire training process, the difference in noise estimation loss between the model equipped with CG-ANI and the baseline model consistently fluctuates around zero, with their 95% confidence intervals largely overlapping. These results indicate that the introduction of CG-ANI does not lead to a systematic reduction in denoising loss, nor does it make the noise prediction objective inherently easier by weakening stochastic perturbations.

Instead, the high statistical consistency in noise estimation difficulty suggests that CG-ANI introduces a confidence-aware adaptive regulation mechanism into the noise injection process while preserving the intrinsic optimization characteristics of diffusion-based reconstruction. By dynamically modulating noise intensity without altering the overall loss landscape, CG-ANI effectively stabilizes the optimization dynamics during training and mitigates excessive fluctuations induced by uniform noise scheduling strategies. This mechanistic interpretation aligns well with the improvements in decision consistency and robustness observed in the ablation experiments—particularly the enhancement in the Kappa coefficient—and provides more solid experimental evidence supporting the effectiveness of the proposed method.

**Figure 7:**
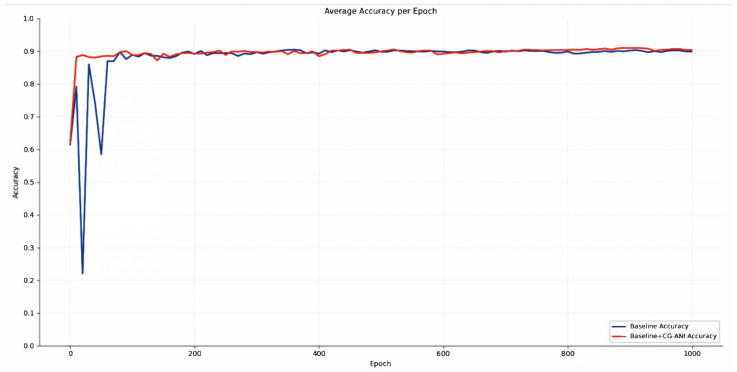
Per-epoch average classification accuracy of the baseline model and the model incorporating CG-ANI, evaluated on the HAM10000 dataset.

In contrast, Figure 7 more intuitively reveals the fundamental differences in training dynamics between the baseline model and the model equipped with CG-ANI. During the early training stage, the accuracy curve of the baseline model exhibits pronounced oscillations, indicating that before stable semantic representations are formed, a fixed noise scheduling strategy tends to impose excessively strong perturbations on discriminative features, thereby leading to optimization instability. This phenomenon does not reflect insufficient discriminative capacity of the model itself, but rather a mismatch between noise injection strength and feature maturity during the diffusion-based training process.

By comparison, after incorporating CG-ANI, the model demonstrates a markedly smoother and more consistent accuracy evolution throughout training. It should be emphasized that this improvement does not stem from a direct enhancement of the discriminative objective or decision boundaries. Instead, CG-ANI adaptively modulates noise intensity based on prediction confidence, effectively suppressing excessive semantic disruption for high-confidence samples in the early training phase, while still preserving sufficient perturbation space for semantically uncertain samples. This adaptive scheduling mechanism enables the model to accumulate meaningful semantic information under a more stable optimization environment.

As training progresses, the accuracies of both models ultimately converge to similar levels, further indicating that CG-ANI does not artificially inflate the final classification performance. Rather, its primary contribution lies in improving training stability and convergence efficiency, allowing the diffusion model to more effectively learn hierarchical and consistent feature representations during the denoising process. From the perspective of optimization dynamics, the results in Figure 7 therefore provide strong evidence that CG-ANI functions as a training stabilization mechanism rather than a direct discriminative enhancement strategy.

Importantly, the observed stabilization effect also provides indirect evidence of the robustness of the proposed framework. The reduced fluctuation amplitude suggests that the training dynamics are less sensitive to stochastic noise perturbations and initialization variability, both of which are common sources of instability in diffusion-based optimization. Moreover, similar performance trends are consistently observed across multiple datasets with diverse imaging characteristics, indicating that the benefits of CG-ANI are not restricted to a specific data distribution or experimental configuration.

Although a dedicated sensitivity analysis is beyond the primary scope of this study, these empirical observations collectively suggest that CGAD-Net does not rely on fragile parameter settings to achieve its performance gains. Instead, the proposed confidence-guided mechanism promotes stable representation learning under heterogeneous conditions, thereby enhancing the overall reliability of the diffusion-based classification process.

## 4. Conclusions

Overall, the experimental results suggest that the proposed CGAD-Net achieves competitive performance across multiple medical image classification benchmarks, including HAM10000, APTOS2019, and Chaoyang, and outperforms the selected comparison methods on several evaluation metrics. These findings should be interpreted with appropriate caution, but they indicate that incorporating structure-aware prior modeling into diffusion-based classification frameworks may enhance discriminative capability and training stability in complex medical imaging scenarios, particularly for tasks characterized by high visual heterogeneity, ambiguous lesion boundaries, or subtle inter-class differences.

From the perspective of network architecture design, the joint introduction of the Hierarchical Pyramid Context Modeling (HPCM) module and the Intra-Scale Dilated Convolution Refinement (IDCR) module provides complementary semantic modeling capabilities for diffusion-based feature learning. Specifically, HPCM alleviates cross-scale semantic inconsistency during the diffusion denoising process through multi-receptive-field contextual modeling, while IDCR focuses on reinforcing structural consistency and local detail representation within fixed-resolution feature levels. The ablation results further confirm that the synergistic interaction between these two modules contributes to the construction of more stable and discriminative feature representations, leading to improved performance and enhanced decision consistency in multi-class medical image classification tasks.

Beyond structural modeling, the proposed Confidence-Guided Adaptive Noise Injection (CG-ANI) strategy also plays a critical role in stabilizing diffusion model training. By dynamically modulating noise intensity according to sample-level prediction confidence, CG-ANI effectively mitigates the mismatch between uniform noise scheduling strategies and heterogeneous semantic reliability across samples, without altering the overall optimization objective. Experimental analyses suggest that CG-ANI does not improve performance through direct enhancement of decision boundaries; instead, it stabilizes training dynamics and reduces excessive semantic perturbations during early training stages, enabling the model to more effectively learn consistent and robust feature representations throughout the diffusion denoising process. This indirect stabilization mechanism contributes to improved training efficiency and supports the observed gains in final performance.

Despite the encouraging results achieved by CGAD-Net, several directions remain worthy of further investigation. First, the soft confidence signal employed in CG-ANI is derived from the classifier’s softmax output and does not constitute a strictly calibrated uncertainty estimate. While this lightweight design avoids additional computational and inference overhead, incorporating uncertainty modeling techniques with stronger theoretical foundations may further improve robustness in highly ambiguous or uncertainty-dominated medical imaging scenarios. Second, the effectiveness of CG-ANI has been primarily validated within diffusion-based classification frameworks using convolutional neural network backbones; its applicability to emerging backbone architectures, such as Transformers or state-space models, remains an open question for future research. In addition, the potential synergy between CG-ANI and more sophisticated sampling strategies or adaptive noise scheduling mechanisms warrants more systematic exploration in subsequent studies.

## Figures and Tables

**Table 1:** Performance comparison on the HAM10000 and APTOS2019 datasets. The results of competing methods are reported from the original publications: MTL [13], LDAM [14], CL [15], ProCo [16], and DiffMIC [28].

Dataset	Metric	MTL	LDAM	CL	ProCo	DiffMIC	Ours
HAM10000	Accuracy	0.811	0.857	0.865	0.887	0.905	**0.914**
	F1-score	0.660	0.734	0.739	0.763	0.815	**0.839**
APTOS2019	Accuracy	0.813	0.813	0.835	0.837	0.838	**0.855**
	F1-score	0.632	0.620	0.652	0.674	0.649	**0.675**

**Table 2:** Performance comparison on the Chaoyang dataset. The results of competing methods are reported from the original publications: Co-tea [17], INCV [18], OUSM [19], HSA-NRL [29], and DiffMIC [28].

Dataset	Metric	Co-Tea	INCV	OUSM	HSA-NRL	DiffMIC	Ours
Chaoyang	Accuracy	0.794	0.803	0.805	0.835	0.868	**0.878**
	F1-score	0.720	0.741	0.737	0.766	0.815	**0.830**

**Table 3:** Component-wise ablation study on the HAM10000 dataset. B0 employs the conventional fixed noise scheduling strategy, whereas B1 isolates the effect of the proposed Confidence-Guided Adaptive Noise Injection (CG-ANI) without introducing architectural modifications, enabling a controlled evaluation of its optimization-level contribution. Higher values indicate better performance.

	CG-ANI	HPCM	IDCR	Accuracy	F1-Score	Kappa
B0	–	–	–	0.905	0.815	0.805
B1	√	–	–	0.910	0.844	0.815
B2	√	√	–	0.912	**0.845**	0.813
**Ours**	√	√	√	**0.914**	0.839	**0.825**

## Data Availability

The HAM10000 dataset used in this study is publicly available at https://challenge.isic-archive.com/data/#2018 (accessed on 15 March 2025). The Chaoyang dataset analyzed in this study is available upon reasonable request from the official project website: https://bupt-ai-cz.github.io/HSA-NRL/ (accessed on 15 March 2025). The APTOS2019 dataset is publicly available at https://www.kaggle.com/competitions/aptos2019-blindness-detection/data (accessed on 15 March 2025).
